# Prosthodontic Management With a Metal Denture Engraved With Laser QR Code

**DOI:** 10.7759/cureus.34483

**Published:** 2023-02-01

**Authors:** Mohammed Mushahed Ali, Shreya Colvenkar, Nashwa Sultana Omer, Shashank Raj Mysolla, Farnaz Noureen

**Affiliations:** 1 Department of Prosthodontics, MNR Dental College and Hospital, Sangareddy, IND

**Keywords:** prosthodontic, forensic, laser qr code, marking, denture

## Abstract

Denture marking is a unique way of identification and is mandated by the global dental council. There are various techniques for marking a denture depending on the prosthesis and method used. In this case report, an elderly patient suffering from Alzheimer’s disease complained of a lack of heat and a cold feeling in the existing denture. The acrylic denture base is replaced by a metal denture and the palatal region is laser sintered with an Aadhar card QR code. This code reveals the patient’s personal details when scanned. This provides quick identification of dentures.

## Introduction

Identity is the most important aspect, not only in the living but also in the deceased. Dental boards all around the globe have guidelines to mark dentures. Denture markings are accepted as a means for keeping records, which is important not only in post-mortem reports but also in medico-legal cases and mass disasters [1].

There are various methods for marking dentures, which are broadly classified into surface-marking and inclusion methods [1-7]. Scribing or engraving, embossing, ID band, laser etching [5], and radio-frequency identification (RFID) tags [4] are commonly used denture-marking methods during routine dental practice.

In countries where there are unique identification numbers for identifying populations and keeping a record, the identification numbers can be used to mark a denture [6,7]. More so, having a QR code that will be linked to such an ID can also be used.

Alzheimer’s disease (AD) is the most common type of dementia and can be defined as a progressive neurologic disorder that causes the brain to shrink (atrophy) and brain cells to die. The early signs of disease are the forgetfulness of events, which progresses to such an extent that the patient is not able to perform even simple tasks [8]. Loss of dentures or misplacing of dentures creates problems for not only the patients but also the caregivers. Hence, denture marking should be considered in such patients.

Metal dentures are manufactured with a medical-grade metal alloy to ensure that denture wearers do not suffer from irritation or allergies. They are not only stronger compared to acrylic dentures but also conduct heat through the denture to underlying tissue, giving a feeling of naturalness while drinking and eating [9,10]. This case report describes a method for marking metal dentures with unique QR codes for a patient with Alzheimer’s disease.

## Case presentation

A 70-year-old male patient presented to the department of prosthodontics with a chief complaint of dissatisfaction with the maxillary denture. On further investigation, the patient revealed that he was a denture wearer for six months and feels a lack of heat and cold sensation with the denture while eating and drinking. The patient wanted to have a natural feel while using dentures.

Medical examination revealed that the patient is suffering from early-stage Alzheimer’s disease. On oral examination, the patient presented with maxillary completely edentulousness and a completely dentulous mandible.

Keeping history in mind, a metal base with a denture marker was planned for the patient. Since the denture base was metal, laser marking with a QR code was planned.

Technique

**Figure 1:**
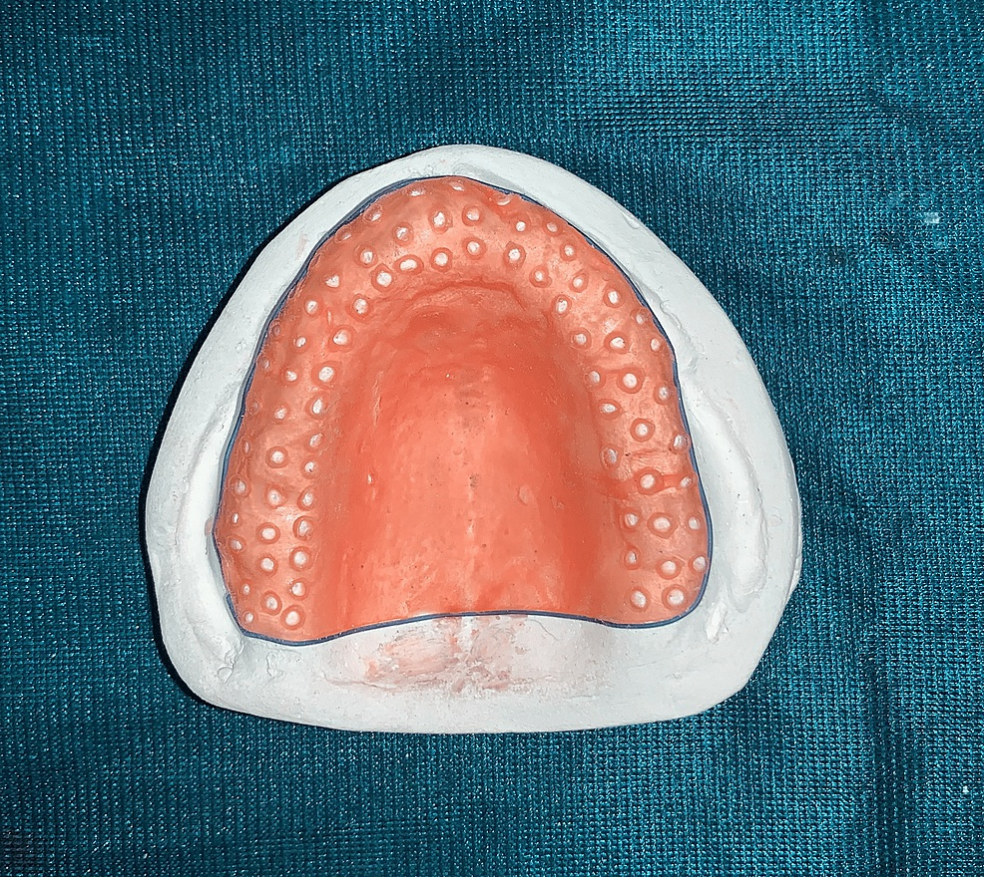
Wax framework for the metal denture base

The primary impression was made with an impression compound, followed by custom tray fabrication, and border molding, and the final impression with a zinc oxide eugenol (ZOE) impression paste. The master cast was poured and a wax pattern for the metal denture base covering the palatal surface and meshwork over the ridge was fabricated (Figure 1).

**Figure 2:**
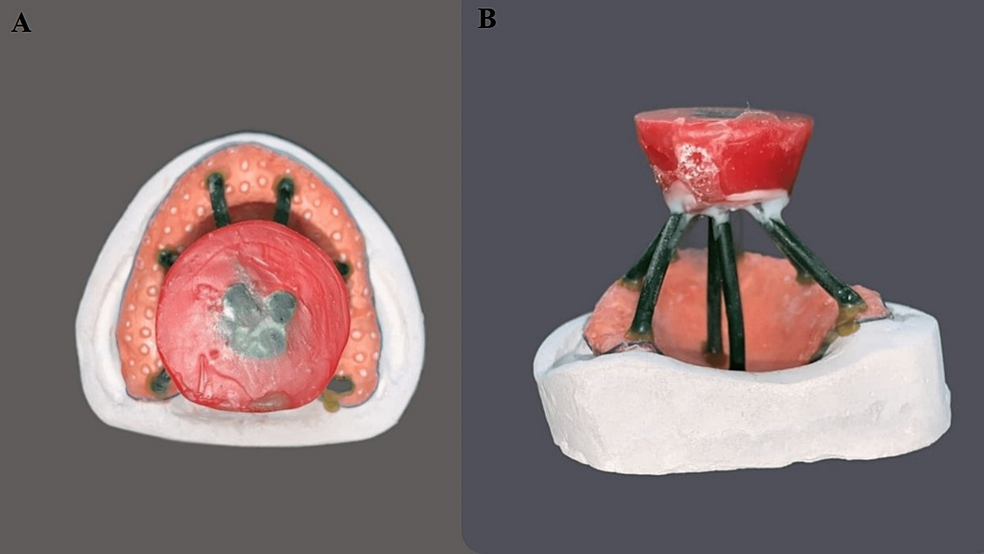
Laboratory procedures A-upper view; B-side view

This was followed by laboratory procedures for casting (Figure 2).

**Figure 3:**
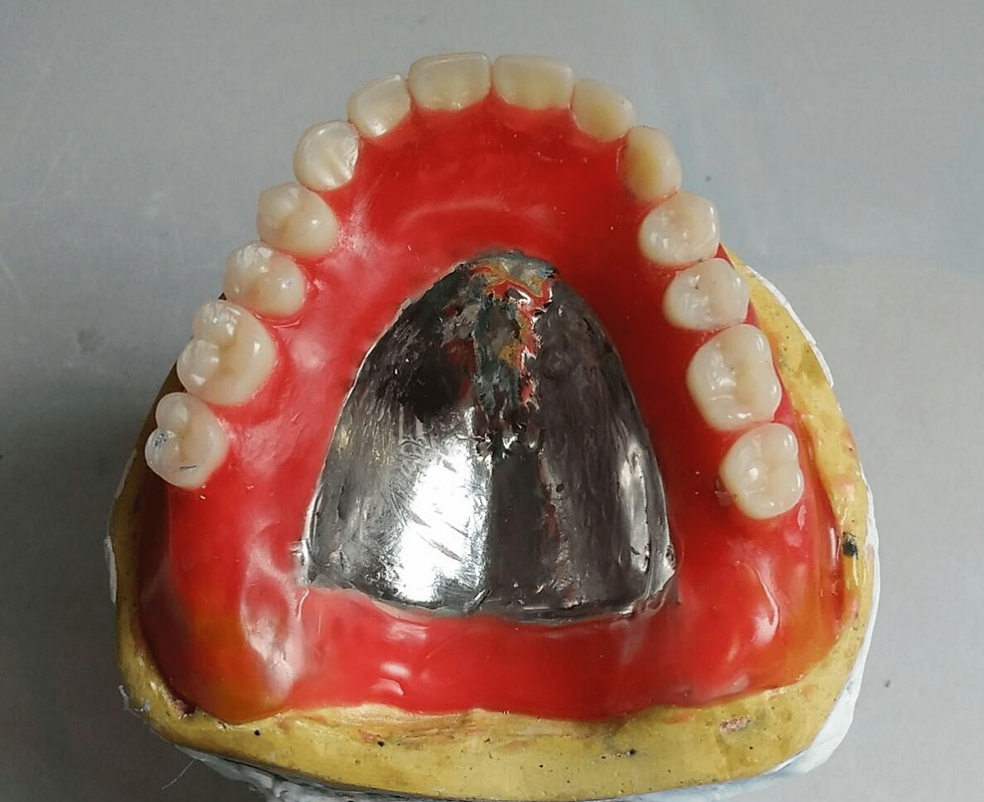
Teeth setting

Over the metal base, a wax occlusal rim was fabricated followed by jaw relations. Teeth arrangement was done according to opposing natural teeth followed by try-in (Figure 3).

**Figure 4:**
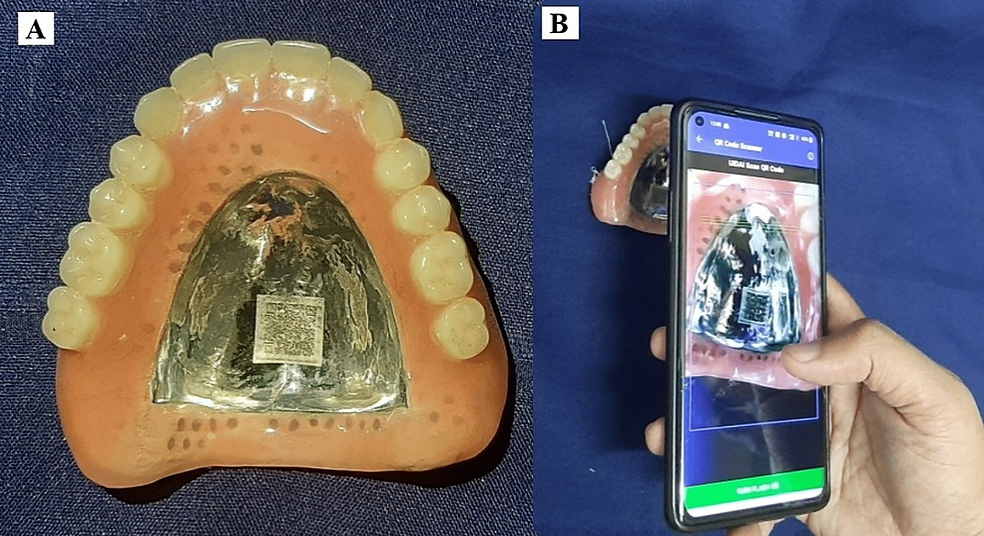
A-metal denture with QR code, B- scanning with mobile

Conventional curing, finishing, and polishing was done. On the metal denture base, the patient’s Aadhar card QR code was sintered using a laser. Scanning of the QR code was done using the Aadhar app and a new window popped up revealing the patient's details on his Aadhar card (Figure 4).

**Figure 5:**
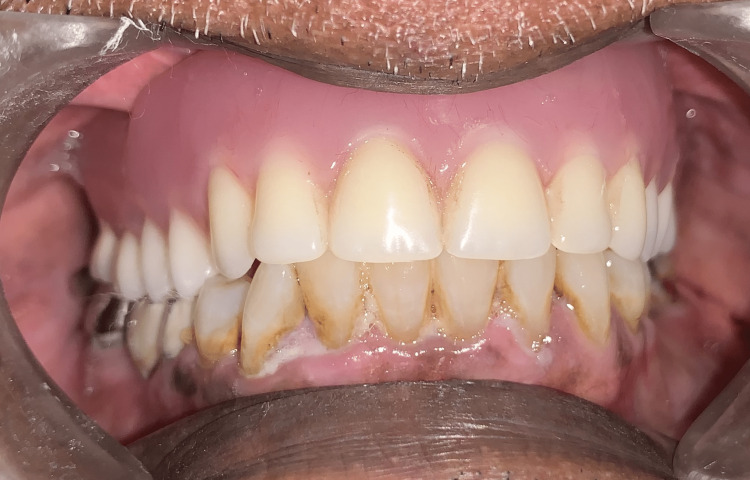
Patient with maxillary denture

The denture was delivered to the patient and post-denture insertion instructions were given to the patient (Figure 5).

## Discussion

In recent times, as we are moving to a well-sophisticated life, patients expect treatment as natural as possible. A heat-cured denture base seems to be better than metal denture bases but has the disadvantage of lack of strength and heat conductivity compared to the metal denture base. Hence, in such cases, especially single denture prostheses, metal usage is recommended [9,10].

Alzheimer’s disease is a progressive neurological disorder that eventually causes the atrophy of the brain. The early signs of disease are the forgetfulness of events that progress to such an extent that the patient is not able to perform even simple tasks [7]. Denture marking is very helpful in such patients, as it saves not only the costs of making new dentures if lost or misplaced but also saves time that patients, as well as caregivers, have to put in. Patel and his colleagues inserted the patient’s photograph in a complete denture patient suffering from Alzheimer’s disease [11].

In this case, the reported patient was dealing with Alzheimer’s disease, and to better handle the symptoms of the disease, it was planned that the prosthesis should be marked so that it can be immediately returned if the patient misplaces or loses in the future. Marking his denture with the QR code of his Aadhar card helps in quick identification, as Aadhar has all the details of the individual. The Aadhaar number is a 12-digit unique identification number that has all patient information such as the iris scan, fingerprints, patient’s age, and address issued by the Unique Identification Authority of India (UIDAI) on behalf of the government of India. The Aadhaar card number scannable by a smartphone will give authenticated and detailed information about the individual. It is inspired by the social security number issued by the United States of America and China.

With regard to the complaint of lack of heat and cold feeling, while having food, a metal denture base in the palatal region and a meshwork on the alveolar ridge region was planned. This not only increased the strength of the denture but also provided warmness to the palatal region while eating or drinking giving a natural sensation to the patient. A review of the literature suggests that metal denture bases are better than acrylic denture bases in terms of thermal conductivity [9,10].

Denture marking is an age-old practice that is hardly practiced by clinicians, though widely recommended by dental boards for forensic reasons. Denture-marking techniques are broadly divided into surface marking and inclusion methods. Surface markers, though economical, can be easily removed by denture cleansers, abrasives, or antiseptic mouthwash. Surface inclusion techniques consist of the incorporation of a marker, with metallic or nonmetallic materials. In the present article, the method to include QR codes stood out from the currently available denture marking methods in various ways. QR code stores a large volume of information, providing rapid access to patients’ information with the use of a smartphone. Denture marking was planned in the palatal region of the maxillary denture as sufficient thickness was present and no technical difficulties were encountered during the placement of the QR code.

## Conclusions

Denture marking is important for patients dealing with progressive neurological disease. There are various methods for marking dentures that entirely depend upon the feasibility of the technique in regard to the type of denture. Since patient expectations were not met from the previous denture, hence metal dentures were fabricated for the patient. Taking into account, the patient’s condition of Alzheimer’s disease, denture marking was done with an Aadhar QR code.
